# Evaluating antimicrobial appropriateness in a tertiary care pediatric ICU in Saudi Arabia: a retrospective cohort study

**DOI:** 10.1186/s13756-020-00842-2

**Published:** 2020-11-03

**Authors:** Yasser M. Kazzaz, Haneen AlTurki, Lama Aleisa, Bashaer Alahmadi, Nora Alfattoh, Nadia Alattas

**Affiliations:** 1 Department of Pediatrics, Ministry of National Guards - Health Affairs, Riyadh, Kingdom of Saudi Arabia; 2 College of Medicine, King Saud Bin Abdulaziz University for Health Sciences, Riyadh, Kingdom of Saudi Arabia; 3 King Abdullah International Medical Research Center, Riyadh, Kingdom of Saudi Arabia

**Keywords:** Saudi Arabia, Children, Days of therapy, Antibiotics, Antimicrobial stewardship, Antimicrobial resistance

## Abstract

**Background:**

Inappropriate antibiotic utilization is associated with the emergence of antimicrobial resistance (AMR) and a decline in antibiotic susceptibility in many pathogenic organisms isolated in intensive care units. Antibiotic stewardship programs (ASPs) have been recommended as a strategy to reduce and delay the impact of AMR. A crucial step in ASPs is understanding antibiotic utilization practices and quantifying the problem of inappropriate antibiotic use to support a targeted solution. We aim to characterize antibiotic utilization and determine the appropriateness of antibiotic prescription in a tertiary care pediatric intensive care unit.

**Methods:**

A retrospective cohort study was conducted at King Abdullah Specialized Children’s Hospital, Riyadh, Saudi Arabia, over a 6-month period. Days of therapy (DOT) and DOT per 1000 patient-days were used as measures of antibiotic consumption. The appropriateness of antibiotic use was assessed by two independent pediatric infectious disease physicians based on the Centers for Disease Control and Prevention 12-step Campaign to prevent antimicrobial resistance among hospitalized children.

**Results:**

During the study period, 497 patients were admitted to the PICU, accounting for 3009 patient-days. A total of 274 antibiotic courses were administered over 2553 antibiotic days. Forty-eight percent of antibiotic courses were found to be nonadherent to at least 1 CDC step. The top reasons were inappropriate antibiotic choice (empirical or definitive) and inappropriate prophylaxis durations. Cefazolin and vancomycin contributed to the highest percentage of inappropriate DOTs.

**Conclusions:**

Antibiotic consumption was high with significant inappropriate utilization. These data could inform decision-making in antimicrobial stewardship programs and strategies. The CDC steps provide a more objective tool and limit biases when assessing antibiotic appropriateness

## Background

Antibiotics are the most common medications prescribed in pediatric intensive care units (PICUs), with up to 50–100% of patients receiving an antibiotic prescription [1–5]. Patients in a PICU setting have a high prevalence of community and hospital-acquired infections and an overall high risk of morbidity and mortality [6–8]. However, antibiotics are not justified in every case and their prescription may signify a lack of judicious use or improper reasoning. It has been found that almost 20–50% of all antibiotics prescribed in a pediatric critical care setting are inappropriate [2, 9]. Such inappropriate antibiotic utilization contributes to emergence of antimicrobial resistance (AMR), adverse drug reactions, and additional morbidity and hospitalization costs [10–12]. It has been estimated that AMR will result in 10 million deaths by 2050 if no global action is taken [13]. In 2015, the World Health Organization (WHO) introduced a global strategy to address AMR that incorporated several interventions, including the reduction of inappropriate antimicrobial utilization [14, 15]. With increasing reports of emerging AMR and declining pathogen susceptibility in ICU patients, Saudi Arabia joined the WHO global action plan for Containment of Antimicrobial Resistance [16–18].

Antimicrobial stewardship programs (ASPs) are recommended to decrease inappropriate antibiotic use and mitigate its effects [19, 20]. This requires an understanding of current antibiotic prescription practices and rationale. Data on antibiotic prescription in our institute have been published without detailing indications, types of infections or the appropriateness of antibiotic prescriptions [21]. Such information helps to quantify the problem and identify the main areas that require attention and modification through an ASP strategy. The objectives of this study were to characterize antibiotic utilization, determine the appropriateness of antibiotic use by applying the Center for Disease Control and Prevention (CDC) 12-step Strategy to prevent antimicrobial resistance among hospitalized children, and study factors associated with inappropriate antibiotic prescription in a tertiary care PICU.

## Methods

### Setting

This retrospective cohort study was conducted at King Abdullah Specialized Children’s Hospital (KASCH), an academic tertiary center in Riyadh, Saudi Arabia with a current capacity of 220 beds. The hospital has a 25-bed closed medical and surgical PICU admitting approximately 1000 patients per year. At the time of this study, there were no approved local antimicrobial guidelines, and no antibiotic stewardship program or strategies were implemented.

### Study design

A retrospective cohort study was conducted from January to June 2017 (6 months). The Institutional Review Board (IRB) at King Abdullah International Medical Research Center (KAIMRC) approved the study, and the requirement for informed consent was waived.

### Population

All pediatric patients aged 0–14 years who were admitted to the PICU and started on antibiotics during the study period were included. Patients were excluded from the study if they were > 14 years of age, or were known to have an immunocompromising condition (including all post-transplant patients and oncological patients within 6 months of receiving chemotherapy), or on immune-compromising medications, or only requiring antifungal or antiviral agents.

### Data collection

Data from electronic medical records were collected using a standardized form and reviewed by two pediatric infectious disease physicians in the study group. Data collected included patient demographics, disease comorbidities, admitting and/or infectious diagnoses, admission type (medical, surgical, trauma, burn), indication for antibiotics, documentation of antibiotic indication in medical chart, microbiological results on documented infections, consultation of infectious disease (ID) services, and antibiotic utilization (type and duration). Identified infections were reviewed, and multidrug-resistant organisms (MDROs) were identified based on susceptibility patterns and resistance to most available antibiotics.

Antibiotic indications were classified as empiric (based on clinical suspicion of infection or positive culture with pending susceptibility), definitive (treating an identified pathogen with known antimicrobial susceptibility), or prophylactic (mainly perioperatively, to prevent infection in a patient at risk) [22, 23]. For empiric antibiotics, decisions about antibiotics after 72 h was classified as follows: antibiotics were stopped (i.e., infection ruled out), antibiotics were continued with a planned duration, antibiotics were changed as per microbial culture results and clinical condition (i.e., de-escalation to definitive treatment), or no action was taken (i.e., no decision or plan was documented and the same antibiotics were continued).

### Outcome definitions

The appropriateness of antibiotic use was independently assessed by two pediatric ID physicians. Consensus agreement was sought in more complex cases and when opinions differed. Appropriateness was based on clinical judgment and classified using the CDC 12-step Campaign to prevent antimicrobial resistance among hospitalized children [24]. The CDC 12-step protocol covers four main domains: preventing infection, effectively diagnosing and treating infection, using antimicrobials wisely, and preventing transmission. Adherence to 5 of the 12 steps relating to the appropriateness of antibiotics was utilized in the study. These steps were targeting the pathogen (step 4), practicing antimicrobial control (step 6), treating infection and not contamination or colonization (step 8), knowing when to say no to antibiotics (step 9) and stopping antibiotics if infection was treated or ruled out (step 10). These steps were elaborated on with some examples to improve clarity and unify understanding of each step for the evaluators (Table 1). Additionally, step 5, accessing the experts (i.e., consulting the ID service), was also evaluated separately.

**Table 1:** Definitions and clarification of selected CDC 12-step recommendations

CDC Step	Clarification
Step 4: Target the pathogen	Inappropriate empiric antibiotic choice based on the likely pathogen Inappropriate definitive antibiotic choice based on identified pathogen susceptibility (need for de-escalation)
Step 6: Practice antimicrobial control	Inappropriate prophylaxis regimen (drug or duration) Inappropriate antibiotic combination (redundant coverage) Inappropriate route of administration (requiring a shift from intravenous administration to oral) Inappropriate dose of antibiotic (based on indication, renal function, etc.) (*not assessed in this study*)
Step 8: Treat infection, not contamination or colonization	Treating contamination or colonization and not a true, lab-confirmed confirmed infection
Step 9: Know when to say ‘no’	Starting empirical vancomycin or broad-spectrum antibiotics (e.g. meropenem, piperacillin/tazobactam, or ceftazidime) unnecessarily
Step 10: Stop infection when cured or unlikely	Continuing antibiotics despite ruling out infection or having negative cultures or completing an appropriate duration of therapy

Days of therapy (DOT) per 1000 patient-days was used to quantify antibiotic consumption. DOT was defined as the number of days that a patient received antibiotics regardless of the dose. When a patient received more than one antibiotic simultaneously, one DOT was counted for every antibiotic given [25]. Patient days were defined as the number of days that all patients were at risk for antimicrobial exposure [26]. Only antibiotics received during the PICU stay were calculated. Any courses prior to or after the PICU stay were not included in data collection or assessed for appropriateness.

### Statistical methods

IBM SPSS version 26 was used to analyze the data. The median and percentile (25th–75th) were used to describe quantitative variables, such as age and duration of antibiotic courses. Frequencies and percentages were used to describe qualitative variables, such as gender, admission type, and indication for antibiotics. Comparisons between appropriate and inappropriate antibiotic courses were performed using Fisher’s exact test for categorical data and Mann–Whitney U test for continuous data. A P-value < 0.05 was considered statistically significant for all analyses.

## Results

During the study period, 497 patients were admitted to the PICU, accounting for 3009 patient days. After excluding 238 patients based on the study criteria, 259 patients were included in the review. Table 2 shows the characteristics of patients in the cohort. One hundred thirty-two (50.8%) patients were male, and the median age was 22 months (IQR: 5–65 months).

**Table 2:** Characteristics of the study cohort

Variable	Entire cohort N = 274		Inappropriate N = 133		Appropriate N = 141		P value
	Count	Percentage	Count	Percentage	Count	Percentage	
Age							
Median months (25th–75th percentile)	22	(5–65)	24	(5–79)	22	(5–56)	0.459
Gender							
Male	132	50.8%	64	50%	68	51.9%	0.804
Female	127	49.2%	64	50%	63	48.1%	
Comorbidities							
Neurologic/neuromuscular	96	35.00%	47	35.30%	49	34.80%	0.510
Pulmonary disease	65	23.70%	23	17.30%	42	29.80%	0.011
Gastrointestinal	50	18.20%	24	18.00%	26	18.40%	0.529
Metabolic diseases	18	6.60%	9	6.80%	9	6.40%	0.545
Endocrine disease	11	4.00%	8	6.00%	3	2.10%	0.091
Cardiac disease	45	16.40%	24	18.00%	21	14.90%	0.294
Renal disease	27	9.90%	15	11.30%	12	8.50%	0.286
Hematological disease	9	3.30%	3	2.30%	6	4.30%	0.280
Preterm	31	11.30%	13	9.80%	18	12.80%	0.278
Genetic/syndromic	74	27.00%	40	30.10%	34	24.10%	0.165
Admission type							
Medical	187	68.2%	75	56.4%	112	79.4%	< 0.001
Surgical	83	30.3%	57	42.9%	26	18.4%	< 0.001
Trauma	3	1.1%	1	0.7%	2	1.4%	0.522
Burn	1	0.4%	0	0%	1	0.8%	0.485
Number of comorbidities							
No comorbidities	99	36.10%	50	37.60%	49	34.80%	0.864
1 to 2	130	47.40%	61	45.90%	69	48.90%	
3 or more	45	16.40%	22	16.50%	23	16.30%	
Type of antibiotic on initiation							
Empiric	187	68.2%	77	57.9%	110	78%	< 0.001
Prophylactic	61	22.3%	45	33.8%	16	11.3%	< 0.001
Therapeutic/definitive	26	9.5%	11	8.3%	15	10.6%	0.323
Indication							
Community acquired pneumonia	53	19.3%	13	9.8%	40	28.4%	< 0.001
Sepsis, hospital acquired	29	10.6%	13	9.8%	16	11.3%	0.411
Sepsis, community acquired	27	9.9%	13	10.5%	13	9.2%	0.436
Bronchiolitis	26	9.5%	14	10.5%	12	8.5%	0.358
CNS procedures e.g., EVD, VP shunt, tumor resection	23	8.4%	19	14.3%	4	2.8%	0.001
Other	116	42.3%	60	45.1%	57	39.7%	0.217
Documented indication in medical chart							
Yes	239	87.2%	117	88%	122	86.5%	0.430
No	35	12.8%	16	12%	19	13.5%	
Appropriate cultures							
Yes	231	84.3%	109	82%	122	86.5%	0.322
No	43	15.7%	24	18%	19	13.5%	
Decision at 72 h for empiric (N = 187)							
Changed	11	5.9%	6	7.8%	5	4.5%	0.364
Continue with planned duration	64	32.2%	25	32.5%	39	35.5%	0.755
No action taken	26	13.9%	16	20.8%	10	9.1%	0.031
Discontinued	86	46%	30	39%	56	50.9%	0.136
Documented infection							
Yes	60	21.9%	29	21.8%	31	22%	1
No	214	78.1%	104	78.2%	110	78%	
MDRO							
Yes	25	41.7%	9	30%	16	53.3%	0.115
No	35	58.3%	21	70%	14	46.7%	
Duration of antibiotics course Median days (25th–75th percentile)	8	5–13	10	6–14	7	4–10	< 0.001

*MDRO* multi-drug resistant organisms

Overall, 259 children received 274 antibiotic courses, resulting in 2553 DOTs. The median duration of an antibiotic course was 8 days (IQR, 5–13). The reason for antibiotic initiation was empiric in 187 courses (68.2%), prophylactic in 61 courses (22.3%), and definitive in 26 courses (9.5%). The most common clinical indications for antibiotic initiation were community-acquired pneumonia (19.3%), hospital-acquired sepsis (10.6%), community-acquired sepsis (9.9%) and bronchiolitis (9.5%).

Compared to appropriate antibiotic courses, inappropriate courses were more likely in surgical admissions and in patients receiving antibiotics prophylactically (P < 0.05). A higher proportion of inappropriate courses occurred in patients receiving antibiotics post CNS procedures (P = 0.001). The duration of antibiotic courses was significantly higher in children with inappropriate antibiotic use than in children who had appropriate antibiotic use (median of 10 vs. 7.0, P ≤ 0.001). For empiric antibiotics, no action taken at 72 h was associated with a higher percentage of inappropriate antibiotic courses, 20.8% versus 9.1% (P = 0.031).

Table 3 shows the distribution of nonadherent courses based on the CDC steps violated. Out of 274 courses, 133 (48.5%) were found to be nonadherent to at least 1 of the five CDC steps. Step 6 (Practice antimicrobial control) was the most prevalent step violated, followed by step 4 (Target the pathogen).

**Table 3:** Inappropriate antibiotics classified by CDC 12-Step

Reason for non-adherence to the CDC 12-step campaign	Number of courses	Percentage
Step 6	48	36.1
Step 4	23	17.3
Step 10	21	15.8
Step 9	6	4.5
Steps 4 and 9	14	10.5
Steps 4 and 10	11	8.3
Steps 6 and 10	1	0.8
Steps 4, 9, and 10	7	5.3
Steps 4, 6 and 9	2	1.5
Total	133 courses	

**Fig. 1:**
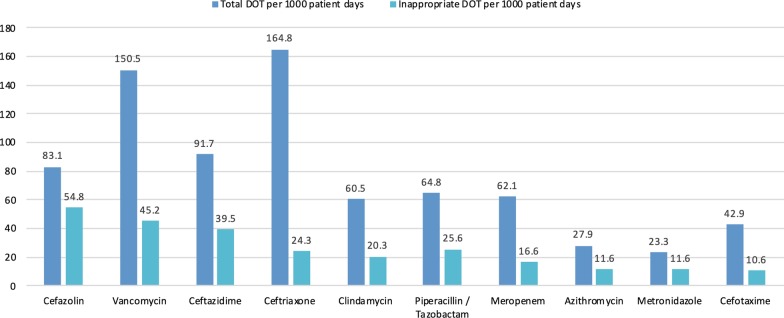
Overall and inappropriate therapy per 1,000 patient days by antimicrobial agent

Figure 1 lists the ten most frequently consumed antibiotics in the PICU and the number of inappropriate DOT per antibiotic. In our center, third generation cephalosporins, vancomycin, and cefazolin were the most frequently used antibiotics. Cefazolin and vancomycin, were the most inappropriately used antibiotic therapies.

## Discussion

The main objectives of this study were to describe antibiotic consumption in our setting, investigate appropriateness of antibiotic utilization by assessing adherence to the CDC’s 12-steps and uncover factors associated with inappropriate antibiotic utilization. The findings suggest a high percentage of inappropriate antibiotics courses. The most common violated CDC steps were “target the pathogen” and “practice antimicrobial control”. There was a higher percentage of inappropriate courses in surgical admissions and with prophylactic courses. Cefazolin and vancomycin had the highest number of inappropriate DOTs.

Total antibiotic consumption was 848.5 DOTs per 1000 patient days, indicating a rising increment from 708.3 DOTs per 1000 patient-days previously reported between 2012 and 2015 [21]. This difference may be attributable in part, to seasonal variation and an increase in prescriptions during winter months in our study period [27]. Our study uncovered a high percentage of inappropriate antibiotic courses (n = 133 out of 274 courses, 48.5%). Previous studies evaluating appropriateness of antibiotics have shown similar percentages in Turkey (50%), Canada (16–60%), and Switzerland (33%) [2, 4, 5].

The most common reason for non-adherence was violation of step 6 (target the pathogen) or step 4 (practice antimicrobial control). Nonadherence to step 4 includes inappropriate empiric or definitive antibiotic choices. Improving empirical and definitive choices would require a better understanding of common pathogens and local susceptibility patterns, and better utilization of diagnostics, in addition to reassessment and taking action after 72 h of antibiotic initiation. Within our cohort, for empiric antibiotics, a lack of action taken at 72 h was associated with a higher percentage of inappropriate antibiotic use (P = 0.031). In view of this finding, conducting an antibiotic ‘time-out’ would be a suitable and effective intervention [26]. A time-out is an ASP intervention recommended by the CDC that standardizes the review of clinical and laboratory results at a predefined time post antibiotic initiation to identify opportunities for discontinuing or deescalating empiric antibiotics [28].

Nonadherence to step 6 suggests an inappropriate prophylactic choice or duration, inappropriate antibiotic combinations, or route of administration. In our cohort 45 out of 61 (74%) prophylactic courses were deemed inappropriate. This may explain why the percentage of inappropriate antibiotics was higher in surgical patients (P < 0.001). Unnecessary prolongation of prophylactic treatment for specific procedures or for the presence of drains may be a contributing factor. Guidelines for perioperative prophylaxis recommend durations not extending beyond 24 h from initiation irrespective of the surgical procedure or the presence of drains or catheters [22, 23].

It is noted that nonadherence to a combination of the CDC steps, and in particular combinations including steps 4, 9, and 10 have contributed to 30.7% of inappropriate DOTs. These steps are related to empiric and definitive antibiotic choices, durations, and avoiding the unnecessary use of broad spectrum agents. Antibiotic treatment decisions in intensive care are challenging and influenced by a difficulty to differentiate between bacterial and viral infections, a lack of gold standard to diagnose pneumonia and an uncertainty in sepsis etiology [29–32]; for instance, in our setting cultures were positive in only 38% of patients who met severe sepsis or septic shock criteria [33]. An international survey on antibiotics decision determinants found significant variability across countries, reflecting cultural and contextual factors [34]. The issue of nonadherence to multiple CDC steps found in the presented study and available literature emphasizes the need to support clinicians and PICU units with adequate diagnostics and local antibiotic guidelines to rationalize choices and durations of treatment.

Cefazolin and vancomycin contributed to one third of inappropriate antibiotic utilization (301 of 801 inappropriate DOTs, 36.6%). The finding that cefazolin had the highest inappropriate utilization supports the finding that CDC step 6 was the most violated step in nonadherent courses and that a higher percentage of inappropriate courses were seen in surgical patients, as it is the drug of choice for peri-operative prophylaxis. Inappropriate vancomycin utilization in our centre corresponds with studies in both adult and pediatric critical care settings [35, 36]. Vancomycin prescription has been increasing in critical care settings, due to the risk of community and hospital acquired methicillin-resistant *Staphylococcus aureus* (MRSA) [37, 38]. Several strategies to control vancomycin use in critical care units have been proposed; negative MRSA surveillance swabs were found to have a high negative predictive value for subsequent MRSA infections [39]. Automating vancomycin prescriptions based on controlled approved indications and vancomycin targeted time-out were associated with reduction of inappropriate vancomycin utilization [26, 35].

This study has several limitations. First, data were collected retrospectively; thus, some information influencing decision making could have been missed. Second, this is a single center report, which limits its generalizability. Third, decisions regarding antibiotic appropriateness are prone to bias, despite assessing the appropriateness in this study by two independent infectious disease physicians based on the unified CDC 12 step classification. Additionally, due to the retrospective nature of the study, the accuracy of diagnoses was not confirmed, and the authors accepted all diagnoses made by treating physicians. This meant that we could not assess nonadherence to step 8 (treat infection but not contamination or colonization).

## Conclusions

We were able to confirm a high prevalence of antibacterial utilization in the PICU with evidence of inappropriate prescription practices, mainly due to inappropriate perioperative prophylaxis durations and inappropriate empiric and definitive antibiotic choices. Based on our findings, the main areas for stewardship interventions include the development of empiric antibiotic guidelines (addresses step 4 and 9), re-education on surgical prophylaxis guidelines (addresses step 6), and the introduction of ‘time-out’ moments post-antibiotic initiation for empiric antibiotics (addresses step 10). Different approaches to antimicrobial stewardship need to be individualized based on identified concerns. Decisions regarding the appropriateness of antibiotic use could be facilitated by the development of local antibiotic guidelines and the utilization of tools such as the CDC steps to both standardize the process and limit bias and subjectivity.

## Data Availability

The datasets used and/or analyzed during the current study are available from the corresponding author on reasonable request.

## Abbreviations

PICU: Pediatric Intensive Care Unit
AMR: Antimicrobial resistance
WHO: World Health Organization
ICU: Intensive care unit
ASP: Antibiotic stewardship programs
KASCH: King Abdullah Specialized Children’s Hospital
KAIMRC: King Abdullah International Medical Research Center
CDC: Centers for Disease Control and Prevention
DOT: Days of therapy
NICU: Neonatal intensive care unit
MRSA: Methicillin-resistant *Staphylococcus aureus*
